# Impact of Probiotic Formula (Lacto-5X) on Constipation: Improvements in Gastrointestinal Symptoms, Gut Microbiome, and Metabolites

**DOI:** 10.4014/jmb.2412.12056

**Published:** 2025-04-09

**Authors:** Yoo-Jeong Jin, Young Jae Park, Jieun Choi, Myung-Soo Kim, Uigi Min, Jonghyun Lim, Jooyeon Kang, Do Yup Lee, Byung-Yong Kim

**Affiliations:** 1 R&D Center, Chong Kun Dang Healthcare, Seoul 07249, Republic of Korea; 2 Department of Agricultural Biotechnology, Seoul National University, Seoul 08826, Republic of Korea; 3 Center for Food and Bioconvergence, Research Institute for Agricultural and Life Sciences, Interdisciplinary Programs in Agricultural Genomics, Seoul National University, Seoul 08826, Republic of Korea; 4 Green Bio Science & Technology, Bio-Food Industrialization, Seoul National University, Pyeongchang-gun, Gangwon-do 25354, Republic of Korea

**Keywords:** Probiotics, constipation, stool consistency, stool frequency, gut microbiome, metabolome

## Abstract

Constipation is characterized by low frequent stools and difficult stool passage. Approximately 16% of the global population experiences these symptoms. Probiotics have shown promise in improving constipation symptoms by modulating the gut microbiome. This study aims to evaluate the effects of a probiotic formula (Lacto-5X) on bowel habits, gastrointestinal symptoms, gut microbiome, and metabolites in adults with mild constipation using a randomized, double-blind, placebo-controlled clinical trial design. At the 4-week endpoint, the Probiotic group had significant improvements in stool consistency, stool frequency, abdominal pain, and straining compared to the Placebo group. Satisfaction with bowel habits and improvement in overall intestinal health were significantly higher in the Probiotic group. Microbiome analysis revealed a significant increase in the abundance of *Lactobacillus* and *L. plantarum* in the Probiotic group at the 4-week endpoint. Metabolome analysis showed that L-proline level in the Probiotic group decreased, while threonic acid level increased at the 4-week endpoint compared to the Placebo group. However, these improvements were not sustained at the 8-week follow-up point. Lacto-5X changes the gut microbiome, leading to changes in metabolites, and it induced improved constipation symptoms. Continuous intake may be necessary to maintain these effects. Further studies are needed to explore the long-term efficacy of Lacto-5X.

## Introduction

Constipation is a gastrointestinal disease in which the stool is infrequent, difficult to excrete the stool, or both. The prevalence of constipation is estimated at 16% worldwide [1]. Constipation occurs more often in women than in men, in older people than in young people, and in Caucasians than in non-Caucasians [2]. It includes a variety of symptoms, such as decreased bowel movements, hard stools, incomplete evacuation, excessive straining, and anorectal obstruction [3]. Laxatives (osmotic or secretory) and bulking agents are mainly used for the treatment of constipation [2]. However, these treatments often fail and may cause side effects such as abdominal bloating and abdominal cramps [2].

Probiotics are defined by the Food and Agriculture Organization of the United Nations (FAO) and the World Health Organization (WHO) as “live microorganisms which when administered in adequate amounts confer a health benefit on the host” [4]. Some studies report that probiotics can improve constipation and its symptoms, such as increasing colonic transit and defecation frequency [5]. Intake of probiotics increased the fecal water content and number of fecal pellets in constipation-induced mice [6]. Additionally, taking probiotics improved abdominal pain in people with constipation in clinical trials [7]. Constipation can be caused by an imbalance in the gut microbiome and can be improved by modulating the gut microbiome. Therefore, probiotics may be a safe and effective treatment for constipation [8]. As probiotics are known to be related to the human microbiome, they are attracting attention for their potential to change the intestinal environment [9].

Microbiome research began in earnest in the late 2000s, and research was mainly conducted on the intestinal microbiome [10]. In the early days, microbiome was considered to simply help with intestinal digestion [11]. However, recent studies have confirmed that changes in microbiome and dysbiosis are correlated with diseases [12]. Additionally, recent studies have reported that there is a correlation between dysbiosis and constipation [13].

Lacto-5X was composed of 5 strains: *L. plantarum* UALp-05, *L. acidophilus* DDS-1, *B. animalis* ssp. lactis UABla-12, *B. bifidum* Bb-06 and *S. thermophilus* CKDB027. Lacto-5X is known to be effective for constipation. In a study that Lacto-5X was administered to rats with loperamide-induced constipation, constipation symptoms such as fecal weight, fecal number, and fecal moisture were improved [14]. However, studies on the effects of Lacto-5X on people with constipation and the mechanisms are still limited.

Therefore, the aim of this study is to clarify the effects of Lacto-5X on improving constipation symptoms, changes in intestinal microbiota and metabolites when administered to people with constipation, and to investigate the correlation among these factors.

## Materials and Methods

### Ethics

This study was conducted from May 2023 through June 2024 at the R&D Center of Chong Kun Dang Healthcare (Republic of Korea). The study protocol was ethically reviewed and approved by the Korea National Institute for Bioethics Policy (KoNIBP) (IRB: P01-202306-06-002). Information about the clinical trial was registered with the Clinical Research Information Service, the Republic of Korea (CRIS; trial number KCT0009347).

### Subjects

Adults with mild constipation were recruited through the website of Chong Kun Dang Healthcare. The inclusion criteria were: 19-69 years old and self-reported stool consistency of type 1-3 on the Bristol Stool Score (BSS). The exclusion criteria were pregnant or intending-to-conceive women, breastfeeding women, those who have consumed dietary supplements or medical products that affect the physiological functions of their intestines, and those who has undergone gastrointestinal surgery.

### Study Design

This study was a randomized, double-blind, placebo-controlled clinical trial. Participants completed online questionnaires through an application (Quantum N.EX.T, Hanmi Science) to assess eligibility and exclusion criteria. Enrolled participants were randomly assigned to either the Probiotic group or the Placebo group using a blocked randomization method, and blinds were maintained throughout the study period. The intervention lasted for 4 week, and compliance was monitored through a daily intake record by the application. During the intervention period, the consumption of probiotics or prebiotics other than the study capsules was prohibited. Questionnaire surveys were conducted through the application. For microbiome and metabolite analysis, fecal samples were collected using fecal collection kits (NBG-1C; NobleBio Inc., Republic of Korea). The fecal samples were collected at the start of the intervention (0-week baseline), at the end of the 4-week intervention (4-week endpoint), and at 8 weeks after a 4-week washout period (8-week follow-up point), for a total period of 8 week from the start of the intervention.

### Probiotic and Placebo Composition

The Probiotic group was administered probiotic capsules (Lacto-5X) that contained 2 × 10^9^ colony-forming units (CFU) probiotics. Lacto-5X was formulated with five probiotic strains, including *L. plantarum* UALp-05 (Chr. Hansen), *L. acidophilus* DDS-1 (Chr. Hansen), *B. animalis* ssp. lactis UABla-12 (Chr. Hansen), *B. bifidum* Bb-06 (DANISCO) and *S. thermophilus* CKDB027 (Chong Kun Dang Bio). The placebo capsules were made up of maltodextrin and had the same amounts of excipients as the probiotic capsules. Both probiotic and placebo capsules were produced and packaged by Suheung Co., Ltd. (Republic of Korea). The probiotic and placebo capsules were identical in appearance and structure. Participants in both groups were instructed to consume one capsule daily for a duration of 4 weeks.

### Questionnaire Survey

To verify bowel habits and gastrointestinal symptoms participants filled out questionnaire surveys using the application (Quantum N.EX.T, Hanmi Science). The BSS was assessed by 7-point illustration scale. Participants indicated stool frequency by choosing one of 8 options: 1 (1 time a week), 2 (2 times a week), 3 (3 times a week), 4 (4 times a week), 5 (5 times a week), 6 (6 times a week), 7 (7 times a week, once daily), 8 (more than 8 times a week). The gastrointestinal symptoms include 7 items (abdominal discomfort, abdominal pain, bloating, gas, incomplete evacuation, straining, urgency), each rated on an 11-point Likert scale (0 to 10). Satisfaction with bowel habits was also rated on an 11-point Likert scale (0 to 10). Improvement in overall intestinal health was estimated by the response (yes or no).

### Microbiome Analysis

The fecal samples were stored at -80°C until further processing. For metagenomic sequencing analysis, genomic DNA was extracted from stool and library construction was performed. Briefly, gDNA was extracted using the Omega pathogen kit (Omega Bio-tek Inc. USA) according to the manufacturer’s instructions. To amplify the V3-V4 regions, sequencing libraries were prepared according to the Ilumina 16S metagenomic sequencing library protocols. The primers used for PCR amplification were forward (5'-TCGTCGGCAGCGTCAGATGTGTATAA GAGACAGCCTACGGGNGGCWGCAG) and reverse (5'-GTCTCGTGGGCTCGGAGATGTGTATAAGAGAC AGGACTACHVGGGTATCTAATCC). Initial denaturation was 95°C for 3 min, amplification was 25 cycles at 95°C for 30 sec, 55°C and 72°C for 30 sec, and final elongation was 72°C for 5 min. The PCR product obtained here was purified with HiAccuBead (AccuGene, Republic of Korea). The purified PCR products were amplified for final library construction using Nextera XT v2 (Illumina, USA), Indexed Primer. All PCR conditions were the same described above, except that the number of amplification cycles was 8 times. After purification the PCR products, they were quantified using Quantus Fluorometer (Promega, USA), and qualified with TapeStation D1000 ScreenTape (Agilent Technologies, Germany). Then, sequencing was performed using the MiSeq platform (Illumina). Removal of adapter sequence and paired-end reads end merging of demultiplexed sequence data were processed, and all sequences outside the 400-600 in length or not merged were excluded. Remaining sequence data were subjected to clustering of operational taxonomic unit (OTU) representative sequences at 98% similarity using NCBI 16S RefSeq records database (https://www.ncbi.nlm.nih.gov/refseq/targetedloci/16S_process/) as reference to determine taxonomic richness after removal of chimeric reads. And OTUs were filtered based on the criterion that at least 3% of their values contained at least one count. All of preprocessing and taxonomic analysis of sequence data were conducted by the pipeline developed in the CLC Genomics Workbench 22.0 (Qiagen, USA) at the R&D Center of Chong Kun Dang Healthcare.

### Metabolite Extraction

Fecal samples (100 mg) were placed in 2.0 ml round-bottom Eppendorf tubes and thawed at 4°C. A stainless steel bead was added to each fecal sample for homogenization. Cold extraction solvent (1.4 ml of methanol: isopropanol:water 3:3:2, v/v/v) was added to each tube. The samples were vortexed for 10 sec to ensure complete mixing, followed by homogenization using a mixer mill at 25 Hz for 90 sec. The mixtures were then sonicated for 10 min on ice and incubated on ice for an additional 30 min [15]. After centrifugation at 13,200 rpm for 10 min at 4°C, 600 μl of the supernatant was transferred to new 1.5 ml Eppendorf tubes for LC-MS analysis, and 40 μl was transferred to 2 ml Eppendorf tubes for short-chain fatty acid (SCFA) derivatization. The aliquots designated for LC-MS analysis were concentrated to complete dryness using a speed vacuum concentrator and stored at -80°C until analysis. Prior to LC-MS analysis, the dried samples were reconstituted with 100 μl of organic solvent (acetonitrile, water, methanol, 70:25:5, v/v/v), sonicated for 10 min on ice, and transferred to polytetrafluoroethylene (PTFE) tubes. The samples were then centrifuged at 9,000 rpm for 5 min at 4°C, and the supernatants were transferred to vials for LC-MS analysis [16].

### SCFA Derivatization Method

For SCFA analysis, the 40 μl aliquots collected during metabolite extraction were derivatized using 3-nitrophenylhydrazine (3-NPH). Specifically, each aliquot was mixed with 20 μl of 200 mM 3-NPH hydrochloride in a 70% acetonitrile solution and 20 μl of 120 mM 1-ethyl-3-(3-dimethylaminopropyl) carbodiimide hydrochloride (EDC·HCl) in a 6% pyridine solution. The mixtures were vortexed for 30 sec and briefly centrifuged for 10 sec [16]. The samples were then incubated at 40°C for 30 min to allow for complete derivatization. After incubation, each reaction mixture was diluted with 1.92 ml of organic solvent (acetonitrile, water, methanol, 70:25:5, v/v/v) to prepare the samples for LC-MS analysis.

### Untargeted Metabolomics

Reverse-phase chromatography was carried out using a Waters Acquity UPLC BEH C18 column (1.7 mm × 100 mm, 1.7 μm) equipped with a Waters Acquity UPLC BEH C18 VanGuard Pre-Column (5.0 mm × 2.1 mm, 1.7 μm) and a Vanquish UPLC system (Thermo Fisher Scientific, USA). For positive ion mode, mobile phases A and B consisted of 0.1% formic acid in deionized water and acetonitrile (ACN), respectively. For negative ion mode, mobile phases A and B consisted of 0.1% acetic acid in deionized water and ACN, respectively. The gradient was programmed as follows: 0-0.1 min, 0.5% B; 0.1-10 min, 80% B; 10-10.1 min, 99.5% B; 10.1-12 min, 99.5% B; 12.0-12.1 min, 0.5 % B; 12.1-15 min, 0.5% B. The flow rate was 0.3 ml/min, the injection volume was 2 μl, and the column temperature was maintained at 40°C [15]. All samples were analyzed in randomized order using a Q-Exactive Focus instrument (Thermo Fisher Scientific) with a heated electrospray ionization (HESI) probe. The system was controlled using Q-Exactive Tune software and Xcalibur 4.0. Full scan spectra were acquired over a mass range of 80 to 1,200 m/z. The AGC target was set to 1 × 10^6^ counts with a resolution of 70,000 FWHM. MS/MS spectra were acquired at a resolution of 17,500 with stepped normalized collision energies of 30, 40, and 50 [17].

### Data Processing

Data processing was performed using MS-Dial (ver. 4.9). Mass tolerances were set to 0.01 Da for MS1 and 0.05 Da for MS2. In the detection parameters, the minimum peak height was set to 10,000 amplitudes, the mass slice width was 0.05 Da, linear weighted moving average smoothing, and a smoothing level of 2 scans. Missing values were imputed by replacing them with half of the minimum detected value for each metabolite across all samples.

### Statistical Analysis

The Wilcoxon test was used to assess bowel habits and gastrointestinal symptoms between the Probiotic group and the Placebo group, and the Chi-square test was used to assess improvement between the Probiotic group and the Placebo group. Statistical analyses were conducted using R statistical software in the RStudio environment, with *p*-values <0.05 considered statistically significant.

Beta-diversity was calculated on the Jaccard distance using the vegdist function from the vegan package (v.2.6-10) in R. The stacked bar plot, core microbiome analysis, DEseq2, and pattern search analysis were created and performed in MicrobiomeAnalyst 2.0 [18]. The bar chart and line plot were generated using GraphPad prism software v.8.0 (GraphPad Software Inc., USA). A pattern search analysis of metabolites was conducted in the Placebo group to differentiate the probiotic supplement-specific effects from those of the placebo using modules implemented in MetaboAnalyst [19]. Using pattern search, metabolites displaying a 1-2-1 (increase at the 4-week endpoint compared to 0-week baseline and decrease at the 8-week follow-up point, or opposite trend) pattern were identified (*p* < 0.1). The univariate statistics analyses in microbiome and metabolome data were performed using R statistical software in the RStudio environment. The following R packages were utilized: the stats package for conducting Student’s *t*-test. The vegan package was used to test possible confounding variables (*e.g.*, age, gender) by conducting permutational multivariate analysis of variance (PERMANOVA). A chord diagram was generated using the R package circlize to visualize significant Spearman correlations (*p* < 0.05, |r| ≥ 0.3) among numeric variables from survey, microbiome, and metabolite datasets. Intra-group links were displayed with lower opacity to de-emphasize within-group associations.

## Results

### Baseline Characteristics

**Fig. 1:**
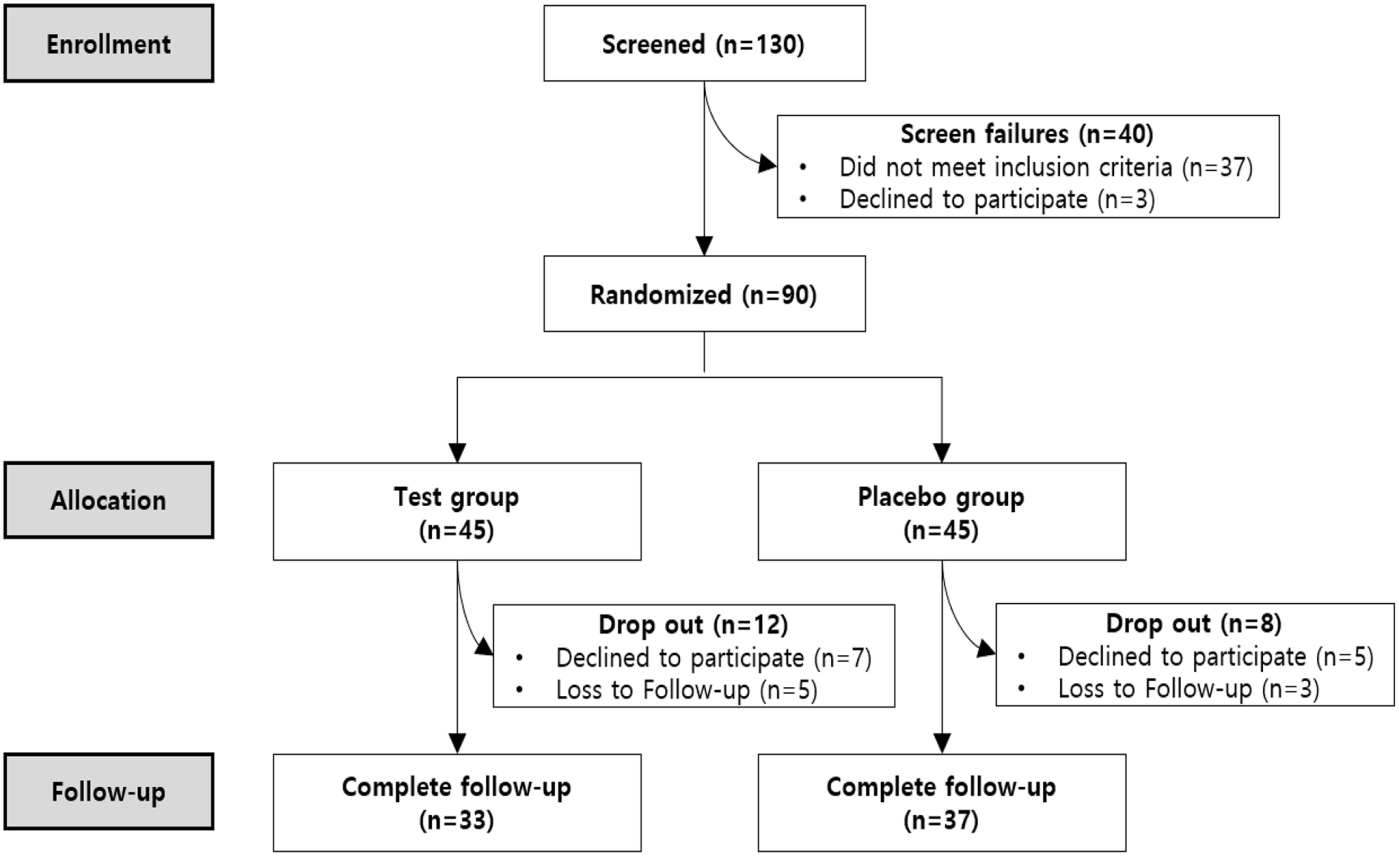
Participant flow chart.

A total of 130 participants aged 19 to 69 years were recruited. Of the recruited participants, 40 were excluded because they did not meet the inclusion criteria or refused to participate. A total of 90 participants successfully enrolled and were categorized into the Probiotic group (*n* = 45) or the Placebo group (n =45) through block-randomized allocation. During the intervention and follow-up period, 12 participants from the Probiotic group and 8 participants from the Placebo group dropped due to declining to participate or being lost to follow-up (Fig. 1). There were no statistically significant differences in clinical characteristics (age, sex), bowel habits, and gastrointestinal symptoms at the 0-week baseline between the Probiotic group and Placebo group (Table 1).

### Bowel Habits and Gastrointestinal Symptoms

**Fig. 2:**
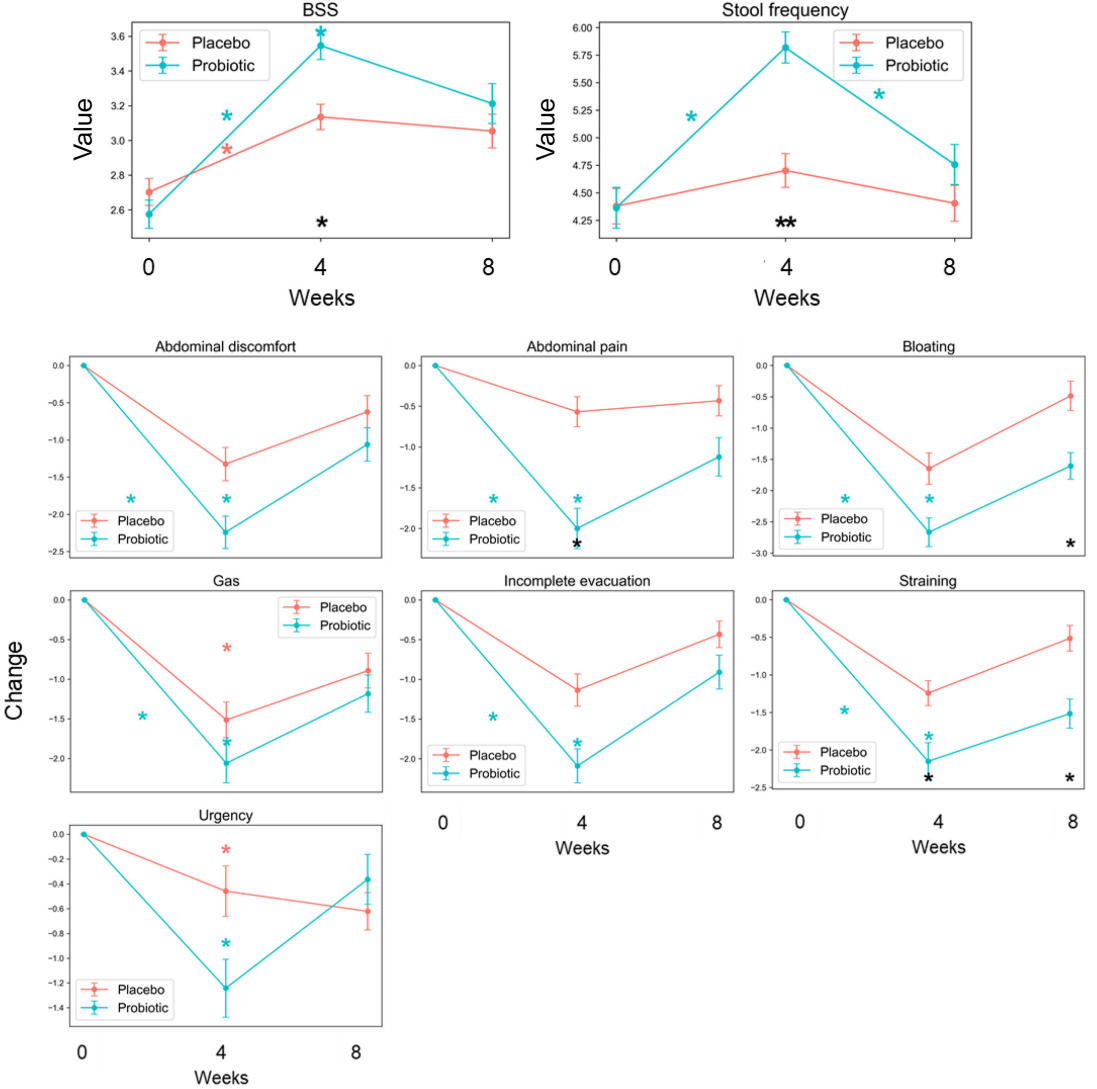
Bowel habits and Gastrointestinal symptoms observed in the intervention by the Probiotic group (Lacto-5X) and the Placebo group. Stool consistency and frequency are expressed by the mean value of measurements (**A**) and gastrointestinal symptoms are expressed by the change from 0-week baseline (**B**) (ns = not significant, * = *p* < 0.05, ** = *p* < 0.01). Black asterisks (*) indicate statistically significant differences between the Placebo and Probiotics groups at the corresponding weeks. Colored asterisks represent statistically significant changes within each group compared to their baseline values. Asterisks (*) are placed within the 0–4, 0–8, or 4–8 week periods based on the corresponding significant time intervals. Error bar means standard error (SE).

At the 4-week endpoint, stool consistency (Assessed using the BSS) was statistically significantly improved (*p*-value = 0.022) in the Probiotic group compared to the Placebo group (3.5 ± 0.9 and 3.1 ± 0.9, respectively) but did not reach significantly at the 8-week follow-up point. Stool frequency also showed a statistically significant increase (*p*-value = 0.006) in the Probiotic group compared to the Placebo group (5.8 ± 1.6 and 4.7 ± 1.9, respectively) at the 4-week endpoint, but no significant increase was observed at the 8-week follow-up point (Fig. 2A).

Among the gastrointestinal symptoms, change in abdominal pain showed significant improvement (*p*-value = 0.016) in the Probiotic group compared to the Placebo group (-2.0 ± 2.8 and -0.6 ± 2.2, respectively) at the 4-week endpoint, but no significant improvement was observed at the 8-week follow-up point. Change in straining also showed significant improvement in the Probiotic group compared to the Placebo group (-2.2 ± 2.9 and -1.2 ± 2.0, *p*-value = 0.033) at the 4-week endpoint and 8-week follow-up point (-1.5 ± 2.2 and -0.5 ± 2.1, *p*-value = 0.016). Change in bloating, significant improvement was shown in the Probiotic group compared to the Placebo group at the 8-week follow-up point (-1.6 ± 2.4 and -0.5 ± 2.9, *p*-value = 0.034). In contrast, no significant differences were observed in the abdominal discomfort, gas, incomplete evacuation, and urgency between the Probiotic group compared to the Placebo group. Gastrointestinal symptoms were assessed by the amount of change from 0-week baseline (Fig. 2B).

**Fig. 3:**
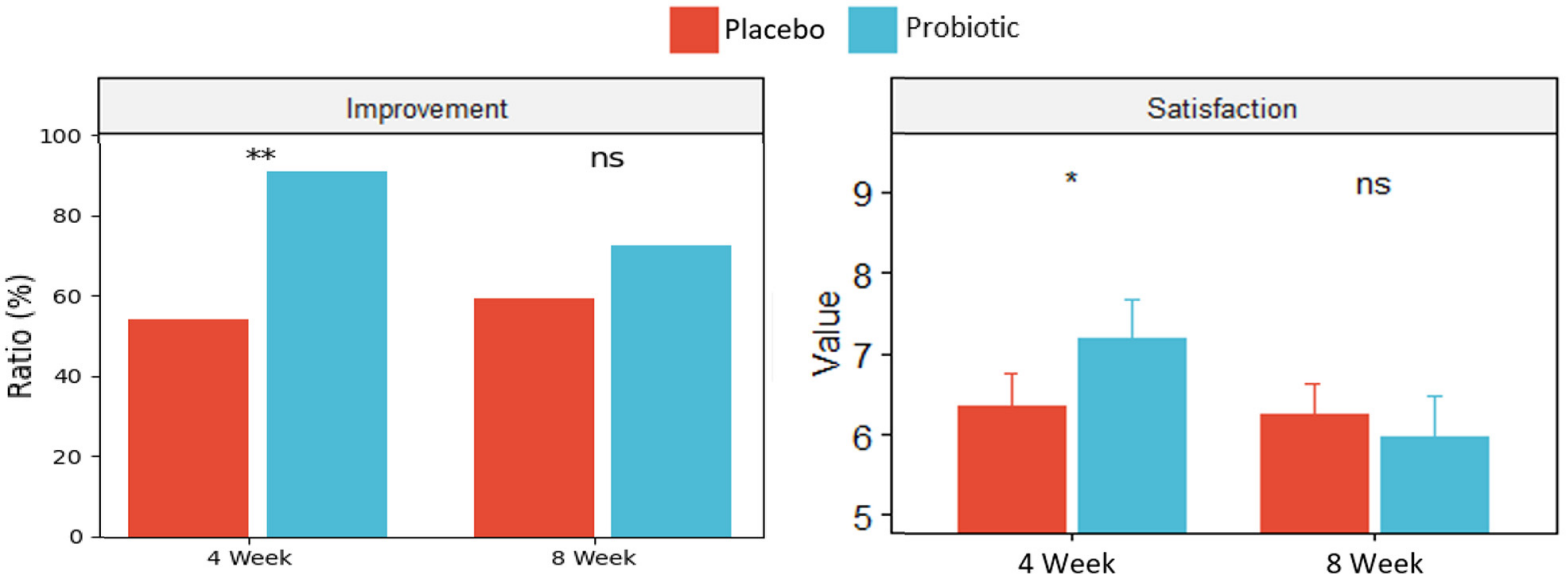
Satisfaction with bowel habits and Improvement in overall intestinal health observed after intervention by the Probiotic group (Lacto-5X) and the Placebo group (ns = not significant, * = *p* < 0.05, ** = *p* < 0.01). Error bar means standard error (SE).

Improvement in overall intestinal was statistically significantly higher (*p*-value = 0.0017) in the Probiotic group compared to the Placebo group (response to ‘yes’ is 91% and 54%, respectively) at the 4-week endpoint, but it was not significant at the 8-week follow-up point. Satisfaction with bowel habits, where higher scores indicate greater improvement, was statistically significantly higher (*p*-value = 0.048) in the Probiotic group compared to the Placebo group at the 4-week endpoint (7.2 ± 2.8 and 6.4 ± 2.5, respectively). However, no significant improvement was observed at the 8-week follow-up point (Fig. 3).

### Core Microbiome Analysis

16S rRNA gene amplicon sequencing data were obtained from human fecal samples, and the gut microbial composition was comparably analyzed between the Probiotic group and the Placebo group at 0-week baseline, 4-week endpoint and 8-week follow-up point. Permutational analysis of variance (PERMANOVA) was applied to test the effect of confounding variables on the microbiome data (Table S1). The microbial taxa were not significantly influenced by host-properties (*e.g.*, gender, age).

**Fig. 4:**
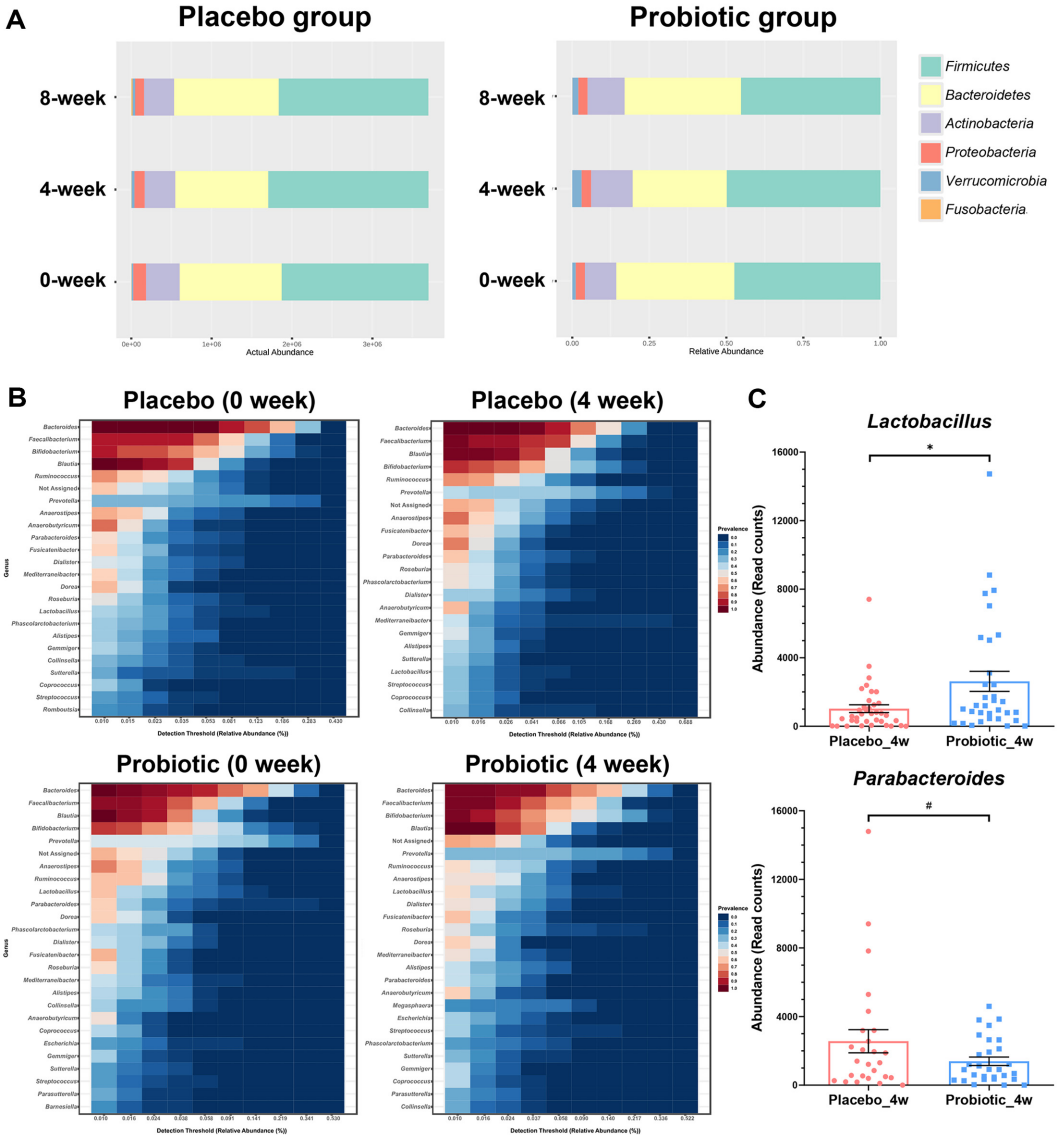
(A) Stacked bar plot of microbial composition at the phylum level. (B) Core microbiome analysis produced in MicrobiomeAnalyst (version 2.0) identifies unique and shared genera for the Placebo group and the Probiotic group based on a minimum 20% prevalence threshold with at least 0.01% detection threshold. (C) Significant differences between the Probiotic group and the Placebo group at 4-week endpoint was estimated based on DEseq2 (# *p*-value < 0.1, * *p*-value < 0.05).

Principal Coordinate Analysis (PCoA) showed no significant discrimination of the OTU compositional profiles across the three timepoints in the Probiotic group (Probiotic group *p*-value = 0.282; Placebo group *p*-value = 0.270)(Fig. S1). Each timepoint in the Probiotic group exhibited different compositions at the phylum level as shown in Fig. 4A. A common difference in both the Probiotic group and the Placebo group was the relatively lower abundance of *Bacteroidetes* at the 4-week endpoint compared to other timepoints. The *Actinobacteria* and *Verrucomicrobia* showed a specific increase pattern at the 4-week endpoint in the Probiotic group.

In the genus level, *Bacteroides*, *Faecalibacterium*, *Bifidobacterium*, and *Blautia* showed the highest prevalence levels across timepoints (0-week baseline and 4-week endpoint) in both the Probiotic group and the Placebo group (Fig. 4C). Notably, only the Probiotic group showed the higher prevalence of *Bifidobacterium* at the 4-week endpoint compared to the 0-week baseline, which was contrast to the pattern in the Placebo group. Furthermore, among the top 25 core microbiome genera at the 4-week endpoint of the Probiotic group, *Lactobacillus* belonging to the phylum *Firmicutes* showed a significant difference at the 4-week endpoint of the Probiotic group compared to the Placebo group (DEseq2 *p*-value = 0.011) (Figs. 4D and S2).

### A Pattern Search Analysis and Differential Expression Analysis

**Fig. 5:**
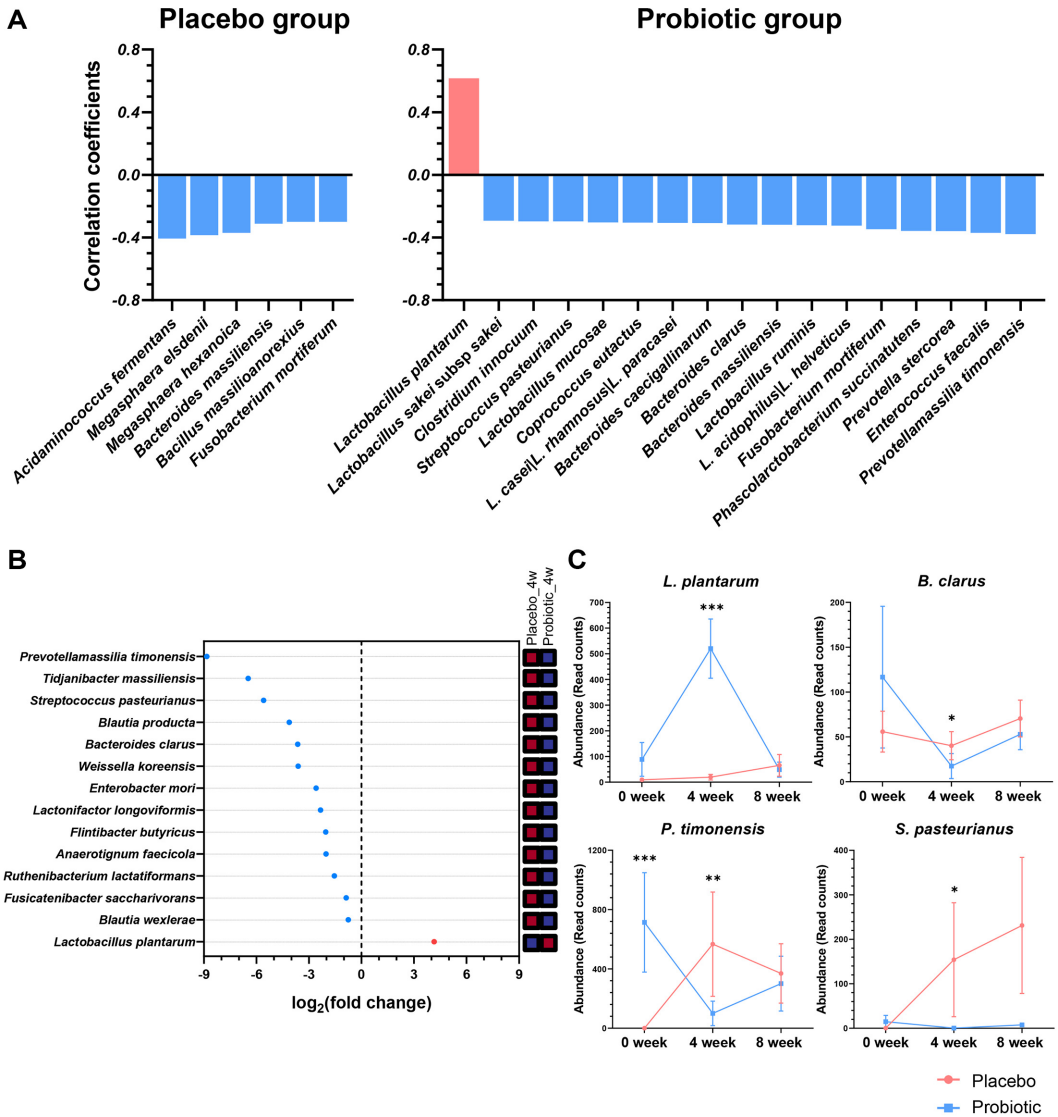
(A) Pattern search using Spearman correlation coefficient analysis of both the Probiotic group and the Placebo group at the species level (FDR < 0.15). (B) Significant difference in species identification between the Probiotic group and the Placebo group by DEseq2 (*p*-value < 0.05). (C) Abundance of the common species. Significant difference between the Probiotic group and the Placebo group at 4-week endpoint was estimated based on DEseq2 (* *p*-value < 0.05, ** *p*-value < 0.01, *** *p*-value < 0.001). The line plot was visualized by GraphPad Prism 8.0.

To identify probiotic treatment-specific effects, we conducted two analyses of the Probiotic group and the Placebo group: (1) pattern search analysis and (2) differential expression analysis using DESeq2. We performed pattern search analysis to identify species that increased at the 4-week endpoint compared to the 0-week baseline and decreased at the 8-week follow-up point, or species that decreased at the 4-week endpoint compared to 0-week baseline followed by increase at the 8-week follow-up point using spearman rank correlation as the distance measure (Fig. 5A). In the Placebo group, all 6 species were negatively correlated with this pattern (FDR < 0.15), showing a decrease at the 4-week endpoint compared to 0-week baseline followed by an increase at the 8-week follow-up point. In the Probiotic group, 16 species also exhibited significant negative correlations, while only *L. plantarum* showed a positive correlation, increasing at the 4-week endpoint compared to 0-week baseline and then decreasing at the 8-week follow-up point (FDR < 0.15). Next, a statistical comparison using DEseq2 was applied to specify the species, focusing on significant differences between the Probiotic group and the Placebo group at the 4-week endpoint (Fig. 5B). Differential expression analysis revealed species-specific changes between the Probiotic and Placebo groups at the 4-week endpoint, with thirteen species significantly downregulated in the Probiotic group and one species, *L. plantarum*, significantly upregulated (*p*-value < 0.05).

To identify species affected by Lacto-5X treatment, we selected the common species from pattern search analysis in the Probiotic group (17 species, FDR < 0.15) and DEseq2 analysis (14 species, *p*-value < 0.05) (Fig. 5C). The 4 species were found in common: *L. plantarum* (pattern search FDR = 5.44E-09; DEseq2 *p*-value = 7.24E-07), *B. clarus* (pattern search FDR = 0.067; DEseq2 *p*-value = 0.027), *P. timonensis* (pattern search FDR = 0.026; DEseq2 *p*-value = 0.002), *S. pasteurianus* (pattern search FDR = 0.087; DEseq2 *p*-value = 0.049). In particular, *L. plantarum* showed significant differential abundance between the Probiotic group and Placebo group, with notably higher levels at the 4-week endpoint in the Probiotic group compared to the 0-week baseline. These findings demonstrate that Lacto-5X treatment induced both (1) distinct temporal patterns at the species level and (2) significant compositional differences compared to placebo, resulting in alterations in microbial species abundance.

### Metabolic Profiling of Human Fecal Samples

**Fig. 6:**
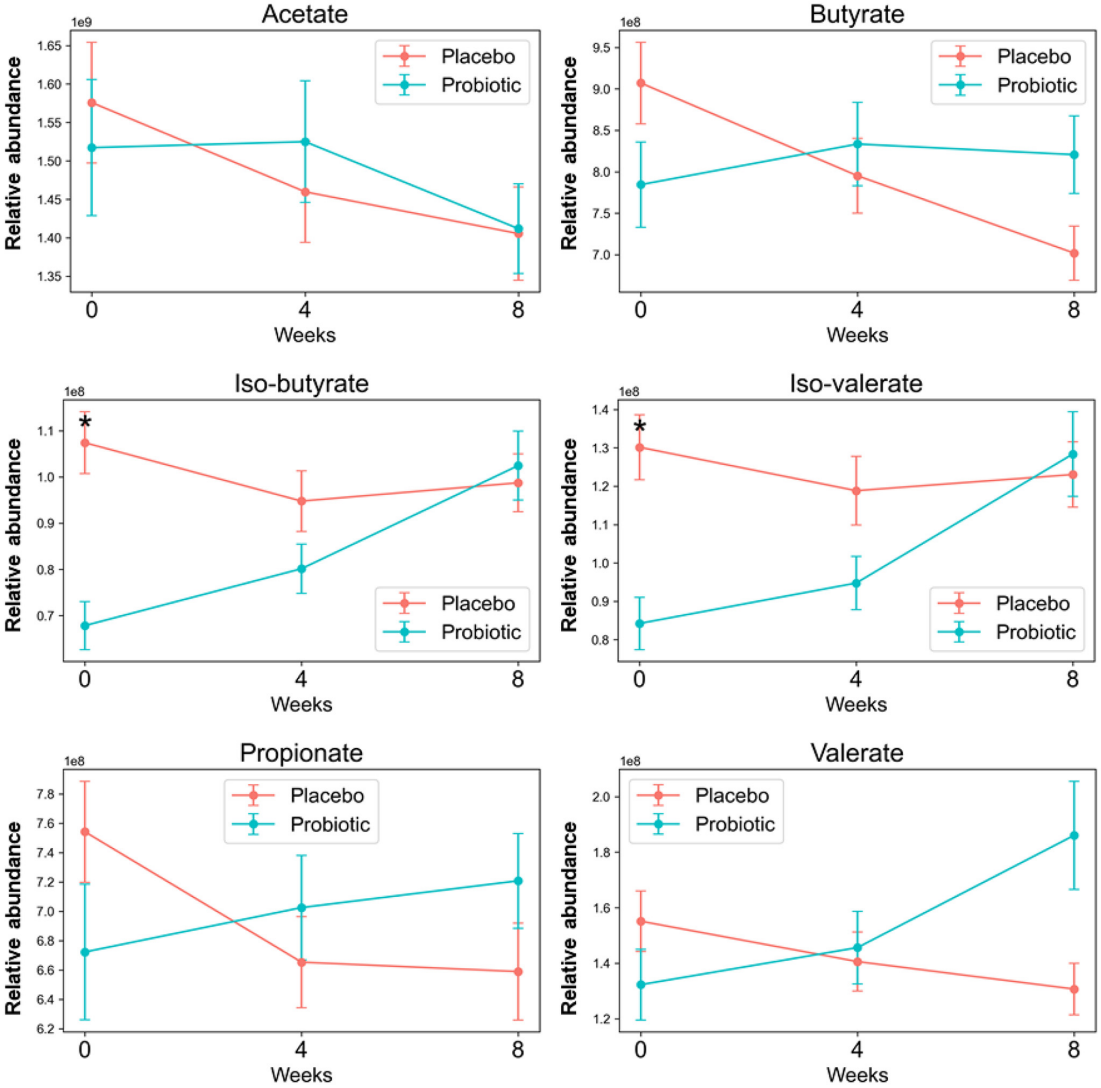
Line plots illustrate the concentrations of six short-chain fatty acids (SCFAs) measured at 0-week baseline, 4-week endpoint, and 8-week follow-up point in the Placebo group and the Probiotic group. Asterisks (*) indicate statistically significant differences between the Placebo group and the Probiotic group as determined by Student’s *t*-test (*p* < 0.1).

To investigate the changes of short-chain fatty acids (SCFAs) in the fecal samples, the content of six SCFAs (iso-butyrate, iso-valerate, valerate, acetate, butyrate, propionate) were measured. There were no significant differences observed in all SCFAs between the Probiotic group and the Placebo group at the 4-week endpoint and the 8-week follow-up point (Fig. 6).

An untargeted metabolomic analysis resulted in 283 metabolites. To identify the probiotic supplement-specific effect, we applied three criteria as follows: 1. Exhibiting the specific temporal pattern; 2. Showing significant changes between 0-week baseline and the 4-week endpoint in the Probiotic group. 3. Displaying the difference between the Placebo group and the probiotic group at 4-week endpoint.

**Fig. 7:**
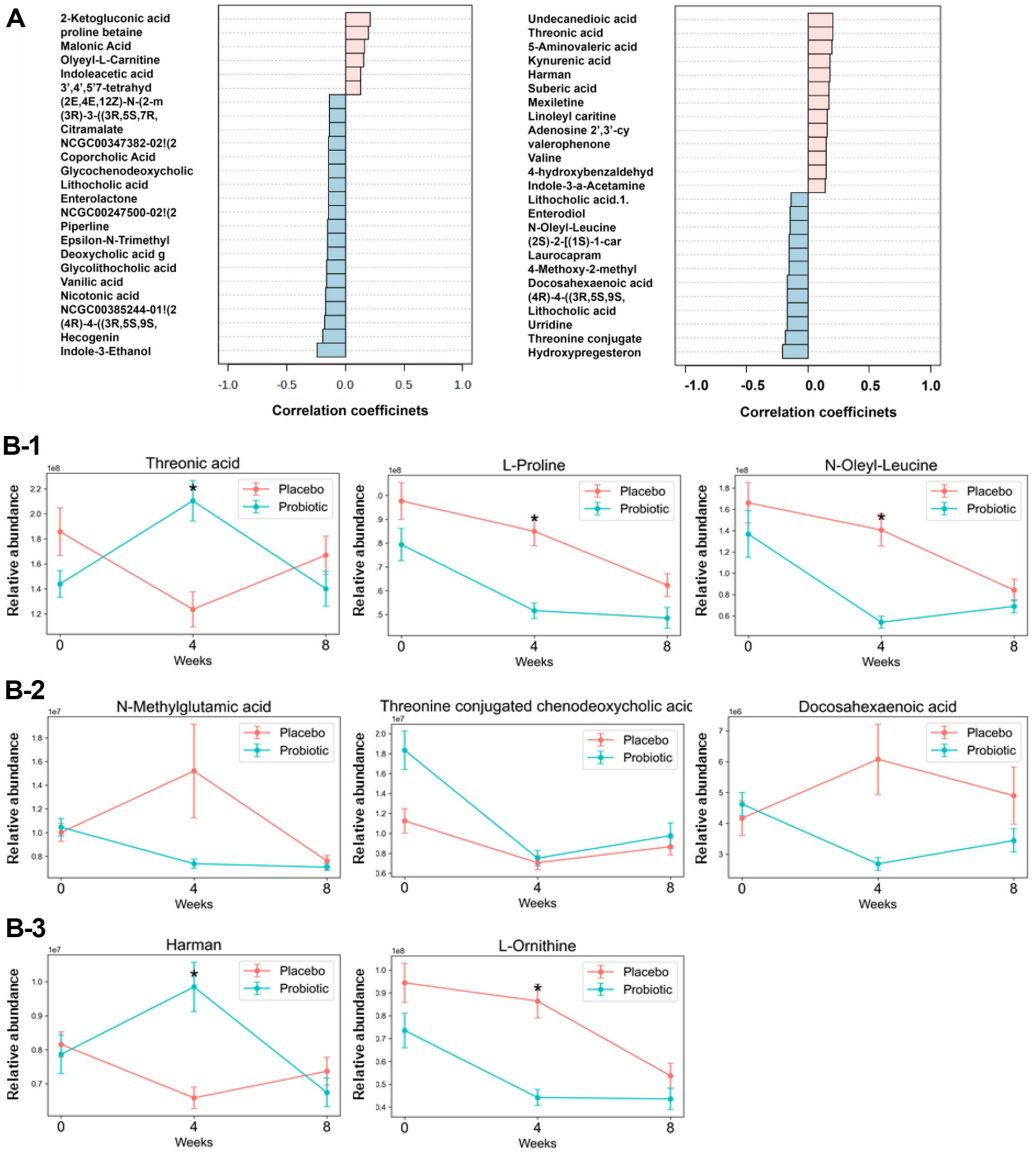
(A) Lists of metabolites exhibiting either an increase at 4-week endpoint followed by a decrease at 8- week follow-up point, or a decrease at 4-week endpoint followed by an increase at 8-week follow-up point in the Placebo (left) and Probiotic (right) groups. (B) Line plots of selected metabolites from the right panel of (B) illustrating changes at 0-week baseline and 4-week endpoint in the Placebo and Probiotic groups. Metabolites are grouped based on three criteria: (i) identified as significant in the pattern search results, (ii) showing a significant difference between baseline (0 week) and 4 weeks within the Probiotic group, and (iii) exhibiting a significant difference between the Placebo and Probiotic groups at 4 weeks. Asterisks (*) indicate statistically significant differences (*p* < 0.1, Student’s *t*-test) between the Placebo and Probiotic groups. B-1: Metabolites that me*et al*l three criteria. B-2: Metabolites meeting criteria (i) and (ii) but not (iii). B-3: Metabolites meeting criteria (i) and (iii) but not (ii).

We conducted a pattern search to identify the specific temporal effects of the probiotic treatment and to differentiate these effects from those of the placebo by performing a separate pattern search in the Placebo group (Fig. 7A). Accordingly, the top 25 metabolites were selected in both the Placebo group and the Probiotic group. The observed differences in temporal changes of metabolites between the Probiotic group and the Placebo group suggest that the probiotic supplement is associated with the specific effects.

Among the metabolites that showed a trend toward statistical significance (*p* < 0.1) in the pattern search results within the Probiotic group, five compounds—threonic acid, L-proline, N-oleyl-leucine, threonine-conjugated chenodeoxycholic acid, and N-methylglutamic acid—exhibited marginally significant differences (*p* < 0.1, *t*-test) between 0-week baseline and the 4-week endpoint in the Probiotic group (Fig. 7B). Additionally, threonic acid, L-proline, N-oleyl-leucine, harman, and L-ornithine showed a trend toward significance (*p* < 0.1, *t*-test) between the placebo group and the Probiotic group at the 4-week endpoint (Fig. 7B). Threonic acid, L-proline, and N-oleyl-leucine m*et al*l these criteria, suggesting they may be potential candidates for probiotic supplement-specific metabolites.

We conducted an integrative analysis to evaluate correlations among survey responses, microbial taxa, and candidate metabolites (L-Proline, Threonic acid, and N-Oleyl-Leucine). Microbial taxa included those with significant differences between the probiotic supplementation and placebo groups at week 4 (*B. clarus*, *L. plantarum*, *P. timonensis*, *S. pasteurianus*, *B. wexlerae*, *F. saccharivorans*, *R. lactatiformans*, *A. faecicola*, *F. butyricus*, *L. longoviformis*, and *E. mori*). A chord diagram showed that L-Proline retained significant negative correlations with abdominal pain, abdominal discomfort, and incomplete evacuation (r = -0.34, -0.31, and -0.39, respectively) (Fig. S3). Threonic acid showed significant negative correlations with both *B. clarus* and *R. lactatiformans* (r = -0.3, -0.38).

## Discussion

This study, we investigated the effects of a probiotic formula (Lacto-5X) intake in 90 adults with mild constipation. Participants were randomly assigned to either Probiotic or Placebo groups for a 4-week intervention period. In this study, Lacto-5X improved gut microbiome, metabolites and gastrointestinal symptoms.

In our study, core microbiome analysis identified a higher prevalence of *Bifidobacterium* at the 4-week endpoint compared to other timepoints in the Probiotic group. Additionally, comparative analysis revealed significantly higher levels of *Lactobacillus* in the Probiotic group compared to the Placebo group. Recent studies have demonstrated that gut microbiota and its metabolites were associated with the development of constipation [20]. And *Lactobacillus* and *Bifidobacterium* have been reported to improve intestinal motility associated with constipation [21]. Moreover, *Parabacteroides*, which was previously reported to be relatively more abundant in constipation patients, showed marginally significant downregulation at the 4-week endpoint in the probiotic group compared to the Placebo group (Fig. 4D) [22].

We identified 17 species that were specifically changed at the 4-week endpoint, implying the treatment-specific effect. *L. plantarum* showed a positive correlation, while the other 16 species exhibited negative correlation patterns. Among the Lacto-5X strains, we detected four species: *L. plantarum*, *B. bifidum*, *S. thermophilus*, and *B. animalis* subsp. *lactis*. (Table S2, Fig. S4). *B. bifidum* and *S. thermophilus* followed a trend similar to *L. plantarum*, while statistical power was low (*p*-value > 0.05). These findings suggest that certain Lacto-5X strains, particularly *L. plantarum*, may transiently colonize the gut but decline post-treatment, highlighting the need for future studies on strain-specific effects and persistence.

Next, the comparative analysis selected 14 species with significantly higher or lower levels, at the 4-week endpoint in the Probiotic group compared to the Placebo group. Among the 4 common species (*e.g.*, *L. plantarum*, *B. clarus*, *P. timonensis*, *S. pasteurianus*), we focused on *L. plantarum* species, which showed distinct changes and different temporal patterns across the timepoints in the Probiotic group compared to the Placebo group. *L. plantarum* was significantly upregulated at the 4-week endpoint compared to the 0-week baseline. Previous studies demonstrated that *L. plantarum* was effective in various disease conditions, such as dyslipidemia, diarrhea, constipation, and inflammatory bowel disease [23]. *B. clarus* was down-regulated at the 4-week endpoint compared to 0-week baseline. This species has been reported to relate to the constipation symptoms and increase the patients' chance of performing a manual evacuation maneuver [24]. These findings suggest that Lacto-5X treatment modulated specific gut microbiota, including increased levels of beneficial species like *L. plantarum* and decreased levels of potentially pathogenic species like *B. clarus*. These changes in the gut microbiota may contribute to an alleviation of constipation-related symptoms.

Short-chain fatty acids (SCFAs) are essential for gut health, serving as energy sources for colonocytes and influencing intestinal motility [25, 26]. Our target analysis showed no significant differences in SCFAs between the Probiotic group and the Placebo group at both the 4-week endpoint and the 8-week follow-up point. Although participants had constipation symptoms, they were otherwise healthy, lacking any chronic or acute diseases. Previous studies have reported minimal effects of probiotics on SCFA levels in healthy individuals [27, 28]. Therefore, our findings align with existing literature, suggesting that probiotics may not significantly affect the SCFA levels in healthy populations, including those with constipation symptoms.

Despite the absence of significant changes in SCFAs, untargeted metabolomic analysis identified probiotic supplement-specific metabolites, including threonic acid and L-proline. In our study involving patients with constipation, supplementation with *L. plantarum*, a component of Lacto-5X, was associated with an approximately 1.5-fold increase in fecal threonic acid levels at the 4-week endpoint compared to baseline. Similar alterations in threonic acid have been reported in a separate study using glioma mouse models treated with probiotics [29]. The recurring observation of threonic acid changes in response to probiotic supplementation suggests that certain gut microbiota-modulating interventions may influence intestinal metabolic profiles. Further investigations are needed to clarify the underlying mechanisms, as well as the clinical significance and potential therapeutic implications of these metabolic shifts for patients with constipation.

L-proline is involved in collagen synthesis and maintains intestinal mucosal integrity [30]. Adequate levels of L-proline are necessary for a healthy gut barrier. However, excessively high dietary intake of L-proline has been associated with elevated serum zonulin levels—a marker of increased gut permeability. This association implies that excessive proline may weaken the gut barrier and facilitate microbial translocation into the bloodstream.

Some bacterial genera, including *Prevotella* and *Prevotellamassilia*, have been linked to metabolic pathways associated with leucine and hydroxyproline, which can serve as proline precursors [31]. In our data, a 4-week intake of probiotics reduced both proline levels and the abundance of *Prevotellamassilia*, specifically *P. timonensis*. These observations are consistent with genus-level patterns that may influence amino acid metabolism. In our study, L-proline levels decreased at the 4-week endpoint compared to 0-week baseline following supplementation with the probiotic strains comprising Lacto-5X. This result suggests that probiotics may help maintain the proper level of proline to prevent the dysfunction of gut barrier, thereby contributing to improved gut health. These findings are consistent with previous research demonstrating that probiotics can enhance intestinal mucosal integrity, thereby contributing to the relief of constipation symptoms [32]. Furthermore, our integrative analysis revealed significant negative associations between L-proline and abdominal pain, abdominal discomfort, and incomplete evacuation (r = -0.34, -0.31, and -0.39, respectively). Threonic acid was also negatively correlated with *B. clarus* and *R. lactatiformans* (r = -0.3, -0.38). These findings highlight the interplay between specific metabolites, gut microbiota, and symptom improvement, suggesting that targeted probiotic interventions could potentially enhance constipation management.

Also, Lacto-5X significantly improved stool consistency, stool frequency, and gastrointestinal symptoms such as abdominal pain and straining at the 4-week endpoint compared to the Placebo group (Fig. 2). Additionally, satisfaction with bowel habits and improvement in overall intestinal health were significantly higher in the Probiotic group compared to the Placebo group at the 4-week endpoint (Fig. 3).

The significant improvement in bowel habits and gastrointestinal symptoms observed in the Probiotic group aligns with previous studies that have reported the beneficial effects of probiotics on constipation. For instance, *L. acidophilus* DDS-1 and *B. lactis* UABla-12, which are included in Lacto-5X, have been shown to improve abdominal pain, bowel habits, stool consistency, and responder rate in IBS patients [33]. An in vivo study with Lacto-5X demonstrated improvements in fecal weight, fecal number, fecal moisture, and gastrointestinal transit ratio in loperamide-induced constipated rat models, although these results were not statistically significant [14]. Additionally, the six strains of probiotics were administered to IBS patients, leading to an increase in the proportion of patients experiencing relief from IBS symptoms [34]. Furthermore, treatment with *B. lactis*, *L. rhamnosus* and fructo-oligosaccharide in patients with functional constipation, as defined by the Rome IV criteria, has been shown to improve the BSS [35].

However, it is noteworthy that these improvements, with the exception of straining during defecation, were not sustained at the 8-week follow-up point. This suggests that the beneficial effects of Lacto-5X on bowel habits and gastrointestinal symptoms may require continuous intake to be maintained. The transient nature of these improvements highlights the potential need for ongoing probiotic supplementation to achieve long-term benefits.

This study has several limitations. First, the intake period of 4-weeks may be insufficient for probiotics to induce significant effect on the intestinal micro-environment. Second, our current study did not control diet, a major factor affecting the gut environment. These limitations may have explained the relatively small effect size by the treatment.

This study demonstrated that Lacto-5X is effective in improving constipation. These results provide evidence supporting the positive impact of probiotics on alleviating constipation. However, long-term investigations are necessary to understand the duration and sustainability of probiotics effects.

Therefore, future studies should aim to explore the long-term efficacy of Lacto-5X while the diet is controlled. These further studies will help us better understand the benefits of customized probiotic prescriptions for people with constipation. In the future, this research could provide a better understanding of the potential benefits of probiotic prescriptions tailored to individuals with compromised intestinal function.

## Supplemental Materials

Supplementary data for this paper are available on-line only at http://jmb.or.kr.



## Figures and Tables

**Table 1:** Baseline characteristics of participants in the Probiotic group (*n* = 33) and Placebo group (*n* = 37)

Clinical parameter		Test (*n* = 33)	Placebo (*n* = 37)	*p*-value
Age (Mean±SD)		45.55±11.43	43.24±11.01	0.36
Sex (Male/Female)		10/23	5/32	0.09
Bowel habits (Mean±SD)	BSS	2.58±0.94	2.70±0.94	0.70
	Stool frequency	4.36±2.13	4.38±1.95	0.97
Gastrointestinal symptoms (Mean±SD)	Abdominal discomfort	3.85±2.59	3.62±2.85	0.59
	Abdominal pain	3.12±3.01	2.49±2.51	0.52
	Bloating	4.52±2.85	3.97±2.91	0.41
	Gas	4.15±2.85	4.22±2.87	0.99
	Incomplete evacuation	3.67±2.75	3.62±2.90	0.86
	Straining	4.27±2.55	3.86±2.79	0.40
	Urgency	3.15±2.65	2.73±2.08	0.60
