# Supplementary material for: Impact of Probiotic Formula (Lacto-5X) on Constipation: Improvements in Gastrointestinal Symptoms, Gut Microbiome, and Metabolites
Source: J Microbiol Biotechnol. 2025 Apr 9;35:e2412056. doi: 10.4014/jmb.2412.12056 (PMC12010067; doi:10.4014/jmb.2412.12056)
Supplement: Supplementary file 1 [file jmb-35-e2412056-supple.pdf]

J. Microbiol. Biotechnol. 2025. 35: e2412056
https://doi.org/10.4014/jmb.2412.12056

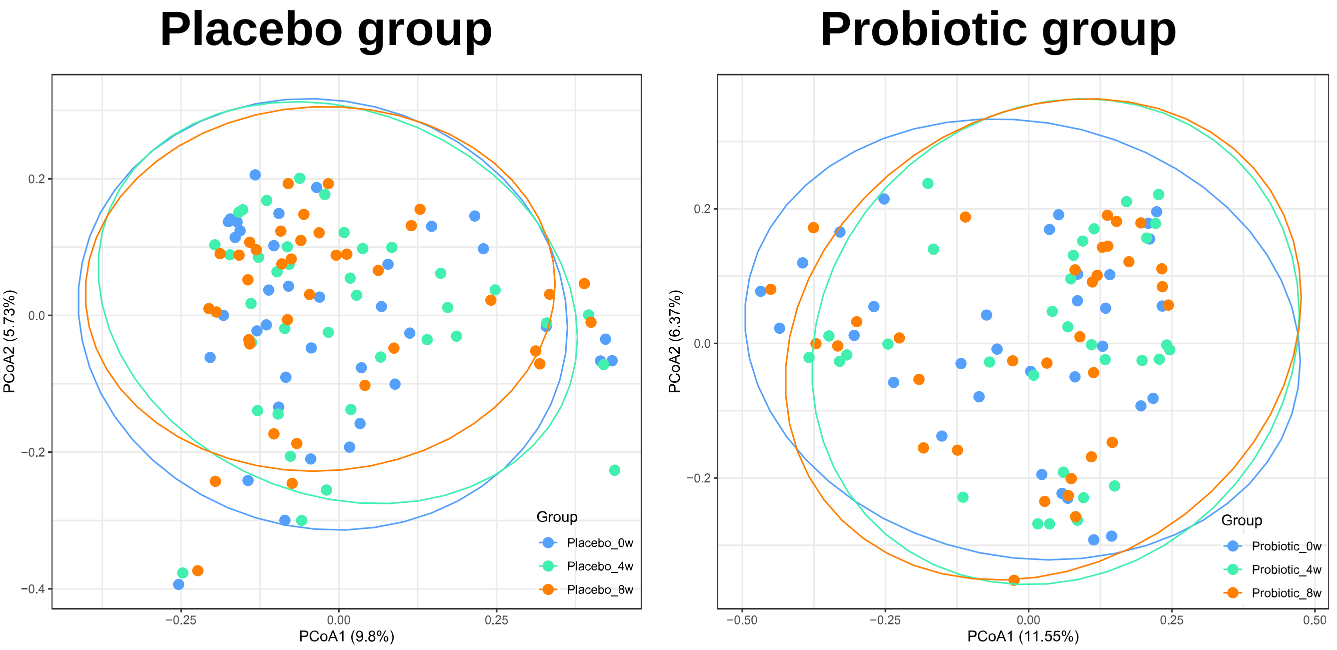

Supplementary Fig. S1 Principal coordinate analysis plots of beta diversity calculated based on the Jaccard index distance method.

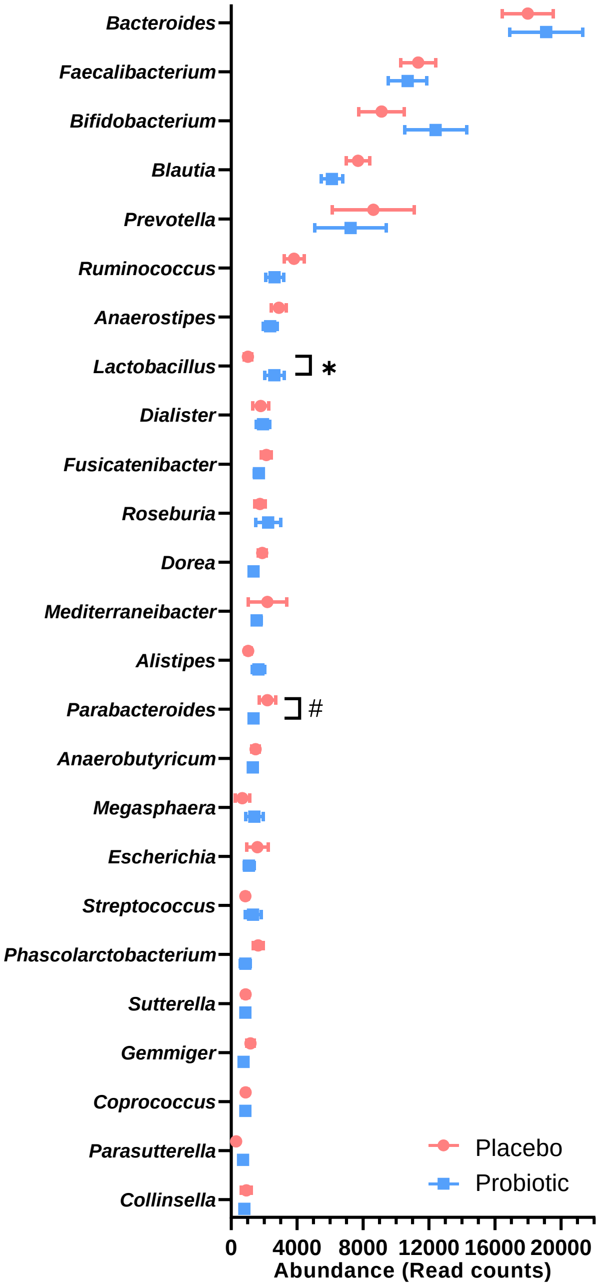

Supplementary Fig. S2 Interleaved plot of microbial abundance at the top 25 genera. The symbols represent the mean value of groups. (circle: Placebo group, square: Probiotic group). The error bars represent ± standard error of measurement (SEM). Significance differences between groups by DEseq2 and p-values are denoted as follows: # p-value < 0.1, * p-value < 0.05.

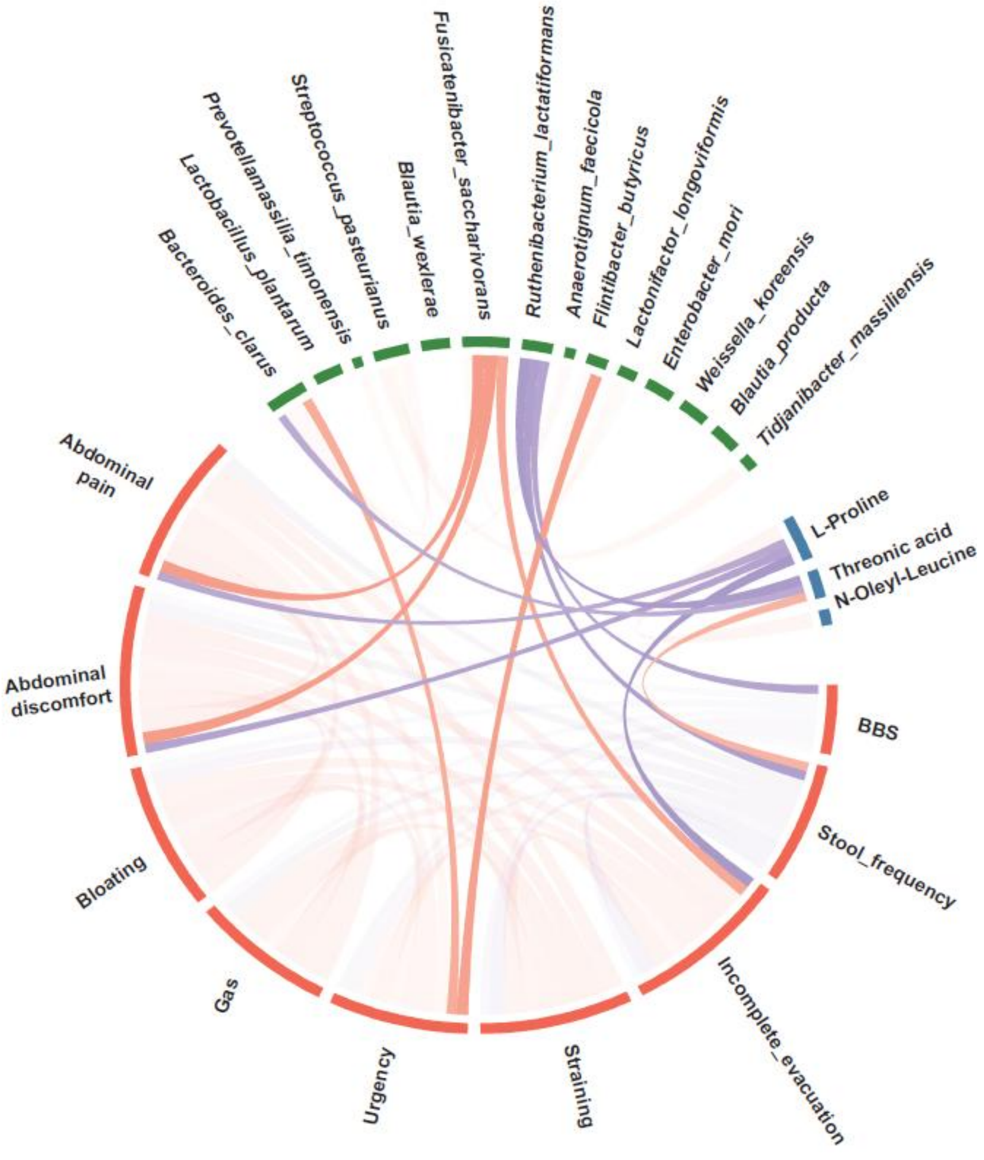

Supplementary Fig. S3. Chord diagram representing the correlations between survey items (red), selected microbial taxa (green), and metabolites (blue). Spearman's correlation coefficients are shown for correlations with coefficients ≥ 0.3 that are statistically significant ($p < 0.05$). Red lines indicate positive correlations, and blue lines indicate negative correlations. Intra-group links are displayed with lower opacity to de-emphasize within-group associations.

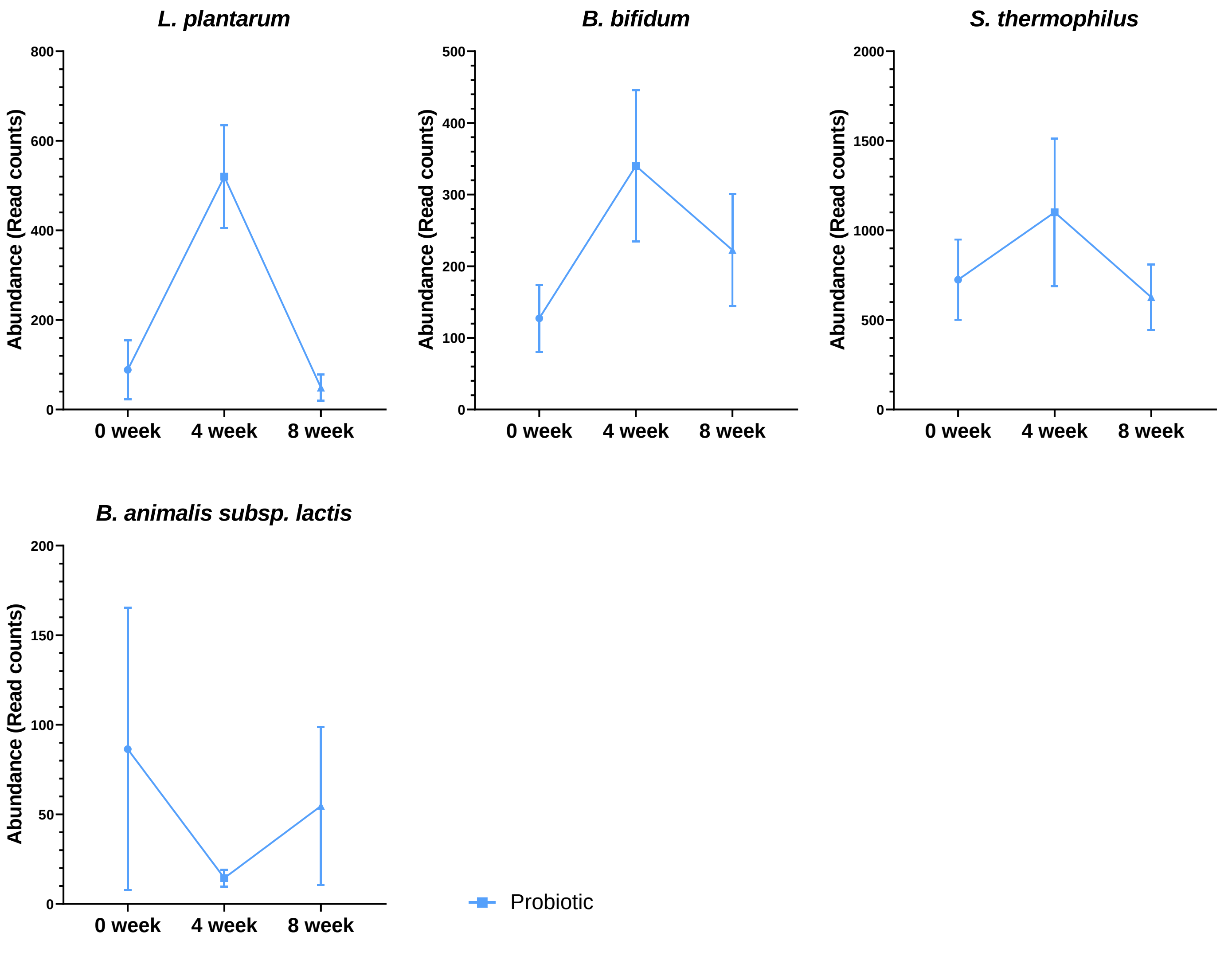

Supplementary Fig. S4 Abundance of Lacto-5X species in the Probiotic group. The square symbols represent the mean value of groups, and the error bars represent ± standard error of measurement (SEM).

Supplementary Table S1. Result of PERMANOVA analysis.

**Microbiome data**		
**Host property**	**R-squared**	**P-value**
**Age**	0.012	0.592
**Sex**	0.022	0.216

Supplementary Table S2. Result of pattern search analysis.

Name	Correlation	FDR
Lactobacillus plantarum	0.616	5.44E-09
Bifidobacterium animalis subsp. Lactis	0.106	0.727
Streptococcus thermophilus	0.081	0.755
Bifidobacterium bifidum	0.010	0.974
